# Rapid complete response of metastatic melanoma in a patient undergoing ipilimumab immunotherapy in the setting of active ulcerative colitis

**DOI:** 10.1186/s40425-015-0064-2

**Published:** 2015-05-19

**Authors:** A Doran Bostwick, April K Salama, Brent A Hanks

**Affiliations:** Departments of Internal Medicine and Pediatrics, Duke University Medical Center, Durham, NC 27710 USA; Melanoma Program, Division of Medical Oncology, Department of Internal Medicine, Duke University Medical Center, 203 Research Drive, MSRB1, Room 397, Box 2639, Durham, NC 27710 USA

**Keywords:** Ipilimumab, Advanced melanoma, Ulcerative colitis, Autoimmunity

## Abstract

While blockade of the cytotoxic T-lymphocyte antigen-4 (CTLA-4) T cell regulatory receptor has become a commonly utilized strategy in the management of advanced melanoma, many questions remain regarding the use of this agent in patient populations with autoimmune disease. We present a case involving the treatment of a patient with stage IV melanoma and ulcerative colitis (UC) with anti-CTLA-4 antibody immunotherapy. Upon initial treatment, the patient developed grade III colitis requiring tumor necrosis factor-alpha (TNF-α) blocking antibody therapy, however re-treatment with anti-CTLA-4 antibody following a total colectomy resulted in a rapid complete response accompanied by the development of a tracheobronchitis, a previously described extra-intestinal manifestation of UC. This case contributes to the evolving literature on the use of checkpoint inhibitors in patients also suffering from autoimmune disease, supports future clinical trials investigating the use of these agents in patients with autoimmune diseases, and suggests that an understanding of the specific molecular pathways involved in a patient’s autoimmune pathology may provide insight into the development of more effective novel combinatorial immunotherapeutic strategies.

## Background

Ipilimumab is a humanized IgG monoclonal antibody that blocks the negative regulatory receptor CTLA-4 on the surface of activated T cells [1]. While ipilimumab has been demonstrated to effectively promote the activation of effector CD8^+^ T cells, recent studies have revealed this agent to also deplete regulatory T cell populations which contribute to the establishment of an immunotolerant tumor microenvironment [2,3]. Almost 25 years after the CTLA-4 gene was cloned, clinical studies showing ipilimumab to significantly prolong overall survival in patients with advanced melanoma earned this agent FDA approval in March of 2011 [4]. While ipilimumab has been associated with a disease control rate of ~25%, treatment with this agent has also been associated with a number of immune-related adverse events including enterocolitis (30-33%), dermatitis (40-43%), hepatitis (3-9%) and autoimmune lymphocytic hypophysitis with anterior panhypopituitarism (1-6%) [5,6]. As evidence that anti-CTLA-4 antibody blockade effectively induces the activation of self-reactive T cells in melanoma patients, studies have associated the induction of autoimmune side-effects with clinical response [7]. Due to the potential for inducing these autoimmune side-effects, there is significant apprehension regarding the use of this agent in patients with underlying autoimmune disease [8]. Given these concerns, there is limited information available regarding the administration of ipilimumab in this clinical setting. A small number of recent case reports have described the administration of ipilimumab to patients with concomitant autoimmune disorders, specifically multiple sclerosis, rheumatoid arthritis, and ulcerative colitis without related exacerbation [8,9]. Here, we wish to add to these reports by describing a patient with known ulcerative colitis who was successfully treated with ipilimumab immunotherapy. While this patient initially developed grade III colitis following his first dose of ipilimumab (3 mg/kg), he later established a rapid complete response to re-treatment with ipilimumab following a total colectomy. In addition, upon re-treatment with the complete four dose regimen of ipilimumab following the colectomy procedure, the patient manifested side-effects consistent with hypophysitis, dermatitis, as well as tracheobronchitis, a condition previously described to occur more frequently in patients with inflammatory bowel disease [10,11].

## Case presentation

**Figure 1:**
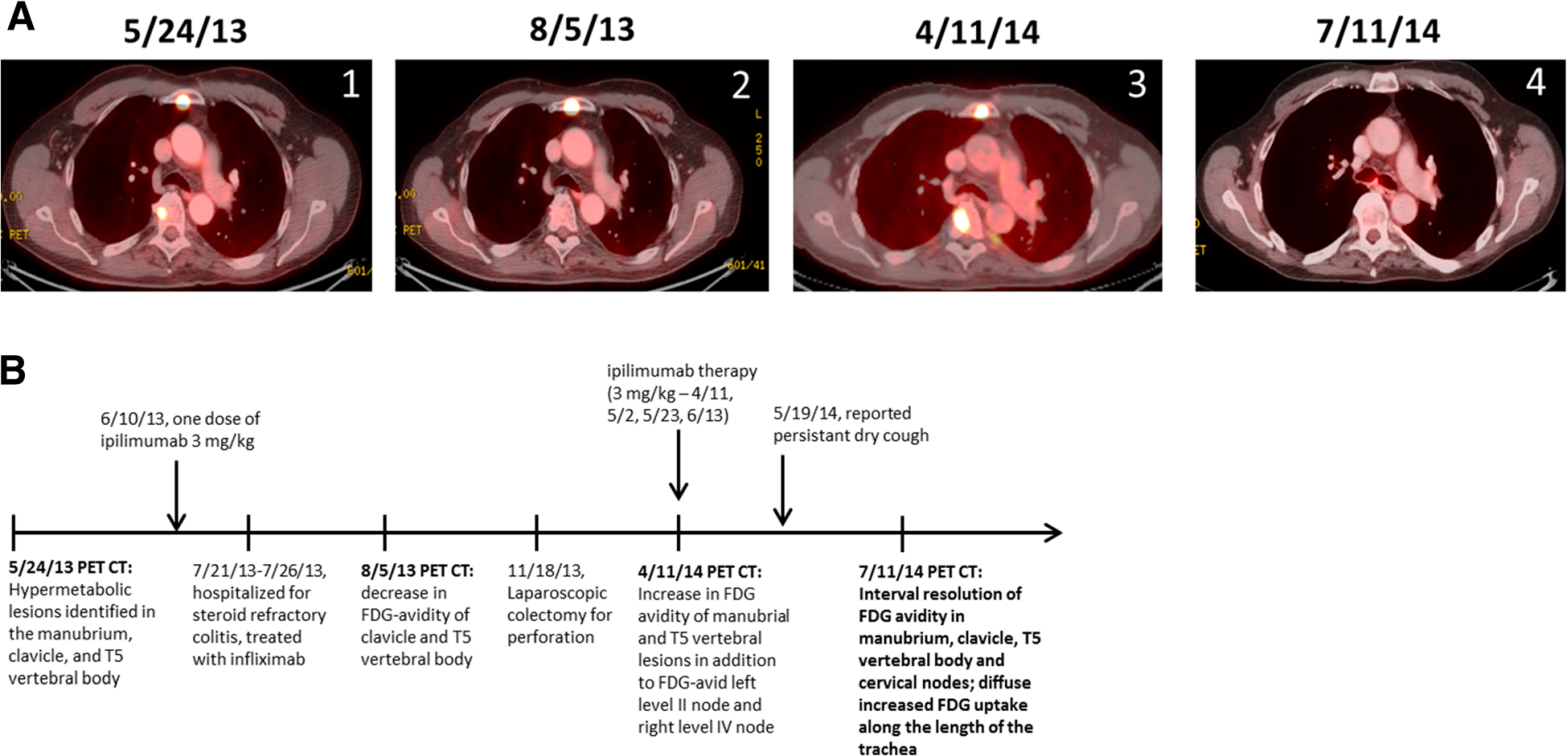
Ipilimumab Treatment Course of Patient with UC. **A**. re-staging chest axial PET CT imaging of patient at different time points. (1) prior to initial dose of ipilimumab, (2) following first dose of ipilimumab and ipilimumab-induced grade III colitis, (3) following total colectomy and prior to re-treatment with ipilimumab, (4) complete disease resolution following a four-dose regimen of ipilimumab. **B**. Time line of clinical events.

**Figure 2:**
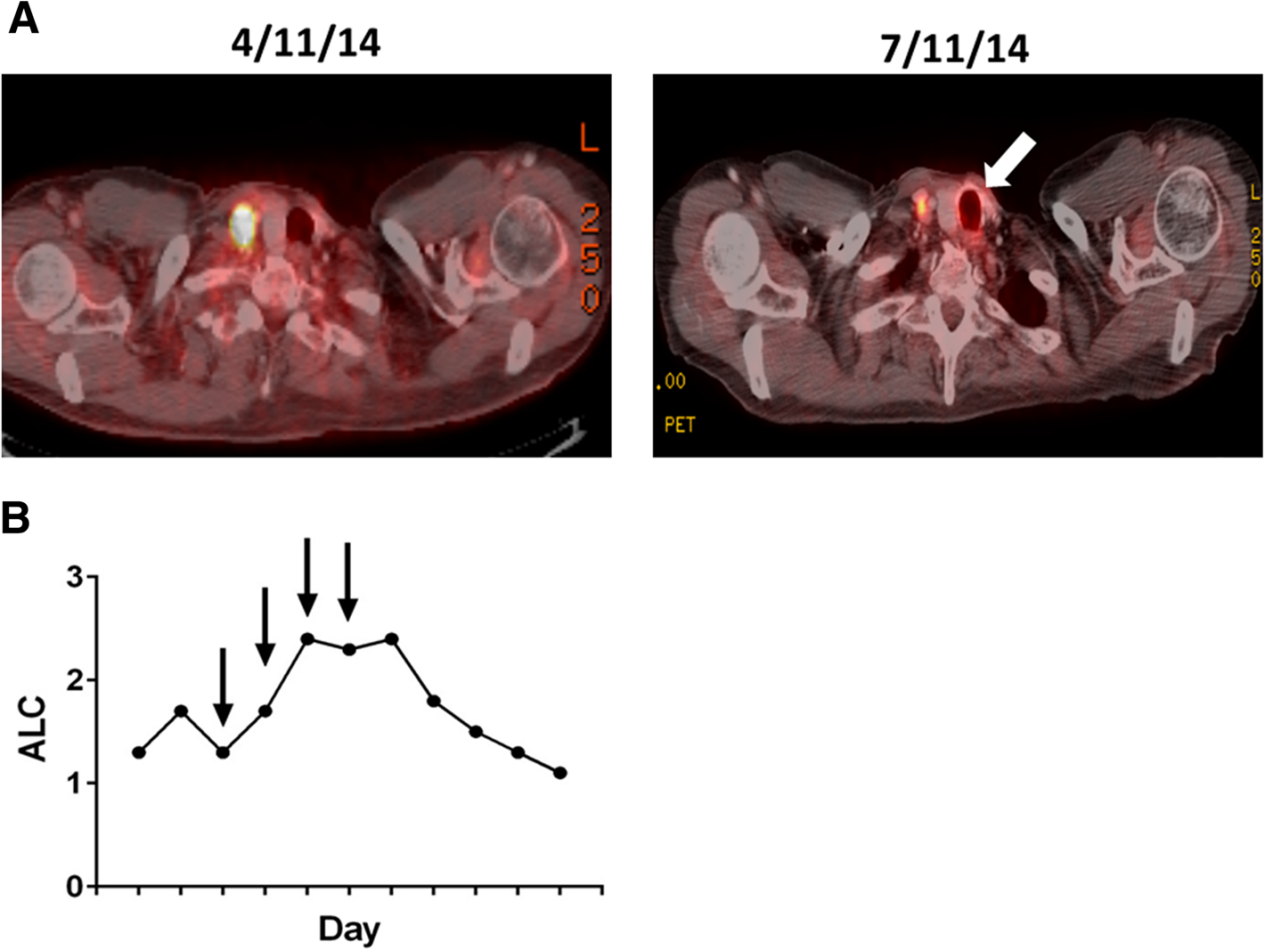
Induction of Tracheobronchitis and Elevation in Absolute Lymphocyte Count Following Treatment of UC Patient with Ipilimumab. **A**. PET CT evidence of ipilimumab-induced tracheobronchitis in a patient with UC (arrow). **B**. Absolute lymphocyte count (ALC) versus treatment day in a UC patient undergoing ipilimumab immunotherapy. Arrows indicate ipilimumab dosing.

A 61-year-old male was initially diagnosed with a melanoma involving his left neck in 1991 and treated with a parotidectomy and neck dissection followed by adjuvant radiation therapy. In July 2009, he was diagnosed with UC, initially treated with infliximab, a chimeric TNF-α monoclonal antibody for 6 weeks and then transitioned to a purine analogue, azathioprine. In May of 2012, he presented with a second primary melanoma involving his left jaw. After a whole body PET revealed no evidence of metastatic disease he underwent a radical neck dissection with pathology revealing invasive malignant melanoma characterized by an ulcerated Clark Level 5 lesion with a Breslow thickness of 5.5 mm and 15 mitotic figures/mm^2^. In August of 2012, PET CT re-staging showed FDG-avid cervical lymph nodes concerning for recurrent disease and a left parotidectomy revealed a 1.8 cm lymph node deposit of malignant melanoma. Surveillance PET imaging in December 2012 demonstrated evidence of further recurrence in bilateral cervical lymph nodes. A right modified neck dissection was positive for 1 of 6 lymph nodes notable for a melanoma deposit up to a diameter of 2 cm. Further surveillance PET CT imaging in May 2013 showed an interval increase in metabolic activity within the lesions in his neck, with additional metastasis to his chest, manubrium, and T5 vertebra (Figure 1A-1). Brain MRI was negative. Given his limited treatment options at the time and after a thorough discussion regarding the potential toxicities in the setting of known UC, it was decided to pursue ipilimumab immunotherapy. Cycle 1 was initiated in June of 2013. Six weeks later he was eventually hospitalized for steroid-refractory colitis characterized by colonoscopy as a moderate colitis with diffuse erosions, ulcerations, and pseudopolyps which resolved with a single dose of infliximab therapy. Further ipilimumab was held and the patient was followed for an additional two months before he was re-admitted for colitis and re-started on azathioprine and IV methylprednisolone followed by a slow prednisone taper in late September 2013. The patient did well until mid-November 2013 when he experienced an episode of acute abdominal pain prompting a diagnosis of colonic perforation and an urgent laparoscopic colectomy. Pathology showed extensive ulcerative colitis with evidence of a sigmoid perforation. Notably, PET CT imaging following his initial hospitalization showed an overall improvement in the FDG avidity of multiple sites of melanoma involving his chest wall as well as various osseous structures including the manubrium, right clavicle, and the T5 vertebral body (Figure 1A-2). After recovering from his surgery and receiving two monthly denosumab injections at 120 mg via subcutaneous delivery, he underwent additional re-staging with a PET CT showing evidence of disease progression (Figure 1A-3). After extensive discussion of potential toxicities including the development of proctitis and/or other autoimmune manifestations involving the hepatobiliary tract such as primary sclerosing cholangitis (PSC), it was determined in April of 2014 that re-induction therapy with ipilimumab in the setting of a total colectomy would be reasonable. He did well until 2 weeks after his second dose of ipilimumab when he reported a grade I limited pruritic rash and persistent dry cough for which he was started on mometasone inhaler and chlorpheniramine/hydrocodone. By five weeks into his ipilimumab regimen, the patient reported the development of a dry cough and two weeks following his fourth dose of therapy, he reported extreme fatigue and a reduction in stamina. Morning labs were drawn and found to be consistent with anterior panhypopituitarism prompting the initiation of hydrocortisone replacement therapy. Although his fatigue improved dramatically, he continued to report a persistent dry cough that was interfering with his sleep. His initial 4 week re-staging PET CT following therapy showed interval complete resolution of prior hypermetabolic FDG activity within the left level II cervical lymph node, the chest wall nodule, and foci in his manubrium, right clavicular head and T5 vertebral body (Figure 1A-4) with diminished FDG activity in a right level IV lymph node. At that time he was also found to have FDG activity along the length of the trachea consistent with a tracheobronchitis (Figure 2A). With this finding, he was placed on a 10 day prednisone taper which completely resolved his cough. He continued on monthly denosumab therapy until he underwent re-staging in October 2014 demonstrating a complete response (CR) to therapy.

## Conclusion

We describe a patient with active ulcerative colitis who underwent treatment with ipilimumab immunotherapy for progressive stage IV melanoma due to limited treatment alternatives. Though the patient experienced a life threatening exacerbation of his ulcerative colitis with administration of ipilimumab, after a total colectomy the patient achieved a marked response to ipilimumab immunotherapy and is, to this date, without evidence of disease. While most patients that ultimately obtain a CR in response to ipilimumab therapy at a dose of 3 mg/kg exhibit relatively rapid objective responses, previous long-term follow-up studies have found patients to require an average of 30 months before obtaining a technical CR [12]. Therefore, this case report describes a rapid response to ipilimumab given the documented CR reported within 6 months of initiating ipilimumab immunotherapy. While the patient never exhibited any signs or symptoms of PSC, he did develop tracheobronchitis and two additional immune-related adverse events (irAE) including a grade I rash and a grade III autoimmune endocrinopathy (anterior panhypopituitarism). The development of a grade III irAE in conjunction with the development of a CR are consistent with prior studies showing a statistically significant association between irAEs and objective response to ipilimumab therapy [12,13]. In addition, this patient also demonstrated a significant increase in his absolute lymphocyte count (ALC), a finding previously noted to correlate with clinical response to ipilimumab (Figure 2B) [14].

The development of a grade IV irAE in a UC patient receiving ipilimumab therapy substantiates previous concerns regarding the use of CTLA-4 blockade in this patient population and emphasizes the need for close clinical monitoring of patients with autoimmune diseases undergoing any form of immunotherapy. This report also raises the concept of a prophylactic colectomy in patients with active inflammatory bowel disease prior to undergoing anti-CTLA-4 antibody therapy. While this approach was discussed following his initial treatment with ipilimumab and repeat hospitalizations for grade III colitis, this was felt to be associated with an unacceptably high risk-benefit ratio given the lack of available data of ipilimumab efficacy in the setting of UC. We acknowledge that this is a single patient experience and is not necessarily applicable to all UC patients that also carry a diagnosis of advanced melanoma. However, given the ultimate outcome of this case, we feel that such an approach should be contemplated on a patient-by-patient basis.

It is tempting to speculate that the altered T cell negative regulatory pathways which have been previously described in UC patients, such as those that modulate the IL-10 receptor signaling pathway, serve to predispose to anti-CTLA-4 antibody-mediated T cell activation and enable a more potent anti-tumor immune response [15]. Whether this patient harbors a genetic mutation that directly impacts effector T cell function is currently unknown. It is also not clear whether patients with specific types of autoimmune disease are more likely to respond to anti-CTLA-4 antibody therapy. Given the various molecular mechanisms that have been identified among patients with the same clinical diagnosis, we hypothesize that patient responses to CTLA-4 blockade are more likely to associate with specific molecular alterations rather than the clinically diagnosed autoimmune disease.

This case report serves to contribute to the limited literature describing the use of an immune checkpoint inhibitor in a patient with an active autoimmune disease and advocates for future clinical trials investigating these agents in this patient population in a controlled setting. We also propose that an understanding of the molecular mechanisms underlying the autoimmune disease of patients who have generated significant responses to anti-CTLA-4 therapy could lead to the identification of novel combinatorial immunotherapy regimens with enhanced clinical efficacy.

## Consent

Written informed consent was obtained from the patient for publication of this case report and any accompanying images. Copies of the written consents are available for review by the Editor-in-Chief of this journal.

## Abbreviations

CTLA-4: Cytotoxic T-lymphocyte-associated antigen-4
irAE: Immune-related adverse event
UC: Ulcerative colitis
TNF-α: Tumor necrosis factor-alpha
ALC: Absolute lymphocyte count
PET CT: Positron emission tomography-computed tomography
FDG: Fluorodeoxyglucose
